# Glucosylsphingosine (Lyso-Gb1) as a reliable biomarker in Gaucher disease: a narrative review

**DOI:** 10.1186/s13023-023-02623-7

**Published:** 2023-02-13

**Authors:** Gaetano Giuffrida, Uros Markovic, Annalisa Condorelli, Valeria Calafiore, Daniela Nicolosi, Marianna Calagna, Stephanie Grasso, Marco Tindaro Valentino Ragusa, Jennifer Gentile, Mariasanta Napolitano

**Affiliations:** 1 Division of Haematology, A.O.U. Policlinico Vittorio Emanuele, Catania, Italy; 2 Oncohematology and BMT Unit, Mediterranean Institute of Oncology, Viagrande, Italy; 3 Department of Biomedical, Dental, Morphological and Functional Imaging Sciences, University of Messina, 98122 Messina, Italy; 4 Postgraduate School of Hematology, University of Catania, Catania, Italy; 5 Sanofi Genzyme, Viale Bodio 37, Milan, Italy; 6 Department of Health Promotion, Mother and Child Care, Internal Medicine and Medical Specialties, University of Palermo, Palermo, Italy

**Keywords:** Biomarker, Gaucher disease, Glucosylsphingosine, Lyso-Gb1

## Abstract

**Background:**

Gaucher disease (GD) is a rare, inherited, autosomal recessive disorder caused by a deficiency of the lysosomal enzyme, acid β-glucosidase. Its diagnosis is achieved via measurements of acid β-glucosidase activity in either fresh peripheral blood leukocytes or dried blood spots, and confirmed by identifying characteristic mutations in the *GBA1* gene. Currently, several biomarkers are available for disease monitoring. Chitotriosidase has been used over the last 20 years to assess the severity of GD, but lacks specificity in GD patients. Conversely, the deacylated form of glucosylceramide, glucosylsphingosine (also known as lyso-Gb1), represents a more reliable biomarker characterized by its high sensitivity and specificity in GD.

**Main text:**

Herein, we review the current literature on lyso-Gb1 and describe evidence supporting its usefulness as a biomarker for diagnosing and evaluating disease severity in GD and monitoring treatment efficacy.

**Conclusion:**

Lyso-Gb1 is the most promising biomarker of GD, as demonstrated by its reliability in reflecting disease burden and monitoring treatment response. Furthermore, lyso-Gb1 may play an important role in the onset of monoclonal gammopathy of uncertain significance, multiple myeloma, and Parkinson’s disease in GD patients.

## Background

Gaucher disease (GD) is a rare, inherited, autosomal recessive, lysosomal storage disorder caused by a deficiency of the lysosomal enzyme, acid β-glucosidase (GBA) (also known as glucosylceramidase and glucocerebrosidase). GBA cleaves glucosylceramide into glucose and ceramide. In GD, a deficiency of GBA functioning leads to the accumulation of glucosylceramide in the lysosomes of macrophages that undergo Gaucher cell transformation. GD occurs in approximately 1 in 450–1,000 live births in individuals of Ashkenazi Jewish descent and has an estimated incidence of 1 in 40,000–60,000 live births in the general population [1]. Furthermore, neonatal screening, combined with a second-tier test to eliminate false positives, identified an incidence of 1 in 16,063 live births in the North-East of Italy [2].

GD is classified according to neurological involvement as non-neuronopathic GD type 1 (GD1) and neuronopathic GD, which is further divided into acute (GD type 2) and chronic (GD type 3) forms [3]. However, disease classification is challenging given the wide spectrum and severity of neurological symptoms [3, 4].

The initial signs and symptoms of GD are often non-specific. However, they can include asthenia due to anemia, bleeding due to thrombocytopenia, platelet aggregation disorders and coagulopathy, abdominal distension due to hepatomegaly and splenomegaly, and bone involvement with painful bone crises, osteopenia/osteoporosis, bone infarcts, pathological fractures, and avascular necrosis. Altogether, GD is a progressive disorder that, if not treated, can lead to severe morbidity due to bleeding, skeletal complications, liver failure, pulmonary hypertension, and sepsis, which negatively impacts patients’ quality of life and life expectancy [5].

The introduction of enzyme replacement therapy (ERT) 30 years ago represented a revolution in the treatment of GD patients. Lifelong ERT is administered intravenously at a dosage between 15 and 120 units/kg every 2 weeks based on disease burden. Significant clinical, laboratory, and radiological improvements occur within the first 6 months, except for irreversible skeletal complications such as pathological fractures and avascular osteonecrosis [6].

Several ERT treatment options are currently available, with no discernable differences identified in their efficacy and safety [7]. These include imiglucerase [8, 9], velaglucerase alfa [10, 11], and taliglucerase alfa [12]. However, taliglucerase alfa is not authorized for use in the European Union [12].

Substrate reduction therapy (SRT), based on glucosylceramide synthesis inhibition and administered orally, also treats the underlying enzyme deficiency in GD and includes the glucosylceramide synthase inhibitor, miglustat [13], and the ceramide analog, eliglustat [14].

Nowadays, however, alternative therapeutic approaches are under investigation, including gene therapy or small molecule glucocerebrosidase chaperones [15].

The rarity of GD, along with its non-specific signs and symptoms, often delays diagnosis, which is determined by measuring GBA activity in fresh peripheral blood leukocytes alongside genetic confirmation of mutations in the *GBA1* gene. However, GBA activity alone is inadequate to assess disease burden at diagnosis, establish treatment criteria, or monitor treatment response. Therefore, GD-specific biomarkers are desirable for improving GD diagnostic rates, assessing disease severity, and monitoring treatment efficacy [16]. In addition, a biomarker must be validated to ensure it is adequate for its intended purpose (see “BEST [Biomarkers, EndpointS, and other Tools] Resource” for comprehensive details on the definition and criteria of a validated biomarker [17]).

Besides ferritin, GD-specific biomarkers commonly used in daily practice include chitotriosidase, chemokine [C–C motif] ligand (CCL18), and the deacylated form of glucosylceramide, glucosylsphingosine (lyso-Gb1, also known as lyso-GL1) [18]. This narrative review evaluates available studies on biomarkers of GD, with a specific focus on lyso-Gb1 in terms of its specificity and sensitivity.

## Materials and methods

### Data sources and search strategy

A literature search was conducted in Pubmed using the following search terms: [Gaucher Disease] AND [Biomarker] AND [Lyso-Gb1 levels] AND [glucosylsphingosine levels] to identify relevant articles, and the reference sections of identified articles were manually screened to identify additional pertinent studies. Studies that met the following eligibility criteria were included in this review: (1) evaluation of biomarkers in GD, with sensitivity and specificity outcomes of interest for GD; (2) role of biomarkers in GD diagnosis, treatment, or prognosis; (3) English language. Reviews and meta-analyses were excluded except to identify relevant studies; however, pooled analyses were included. Due to the heterogeneity of available studies, the results of this review are summarized narratively.

## Results

### Biomarkers associated with GD

As a biomarker for GD, chitotriosidase has been utilized for more than 20 years since the first description of chitotriosidase release by Gaucher cells in 1994 by Hollak and colleagues, who identified elevated chitotriosidase activity in > 90% of symptomatic GD1 patients [19]. Furthermore, chitotriosidase, rather than acid phosphatase or angiotensin-converting enzyme (ACE), was the preferred biomarker of treatment response in GD patients treated with ERT [20].

Chitotriosidase is considered a valid GD biomarker due to its assay’s wide availability and sensitivity. It is part of the primary diagnostic process for the assessment of disease progression and, given the rapid decline in plasma chitotriosidase levels with both ERT and SRT, is used for treatment monitoring and management [21]. However, elevated chitotriosidase activity also occurs in other lysosomal storage disorders and inflammatory processes (i.e., tuberculosis, sarcoidosis, and β-thalassemia, Krabbe disease, GM1 gangliosidosis, Nieman-Pick disease) due to macrophage activation, which compromises its specificity, although chitotriosidase levels are highest in GD patients [22–24].

Notably, chitotriosidase serum levels within the normal range were identified in two symptomatic GD patients [19], highlighting a further limitation of chitotriosidase as a biomarker. Indeed, genome sequencing of the chitotriosidase gene, *CHIT1*, confirmed the presence of a 24-base pair duplication (c.1049_1072dup24 polymorphism), which is present in homozygosity in approximately 6% of the world’s population and causes a deficiency of plasma chitotriosidase activity [25–28].

The chemokine, CCL18, is an alternative option for disease monitoring in patients with chitotriosidase deficiency [21, 29] and was demonstrated to be released by Gaucher cells, highlighting its potential as a biomarker to monitor GD progression [30].

Tartrate-resistant acid phosphatase 5b (TRAP5b) is secreted by osteoclasts reflecting their activity during bone resorption and may be a clinically relevant biomarker of skeletal manifestations in GD patients [31]. Macrophage inflammatory protein 1β (MIP-1β or CCL4) is also used as a skeletal biomarker in GD. Plasma MIP-1β is generally used to monitor multiple myeloma skeletal lesions, although van Breemen and colleagues demonstrated high levels of MIP-1β in patients with GD [32]. The increase in plasma levels of MIP-1β was associated with bone manifestations over the course of GD, with a substantial decrease in plasma MIP-1β levels observed during ERT, except in patients with ongoing skeletal disease.

ACE released by activated splenic macrophages differs from that produced by hepatic macrophages and dendritic cells in sarcoid granulomas; thus, conformational differences in ACE may be adopted as a specific biomarker for GD [33].

However, as described in Table 1, these biomarkers reflect a secondary disease abnormality as an epiphenomenon of macrophage activation. They are thus not directly involved in the pathology of GD. Therefore, identifying a specific biomarker for GD is mandatory to optimize patient management.

**Table 1:** Biomarkers associated with Gaucher disease

References	Study design	Population	Key findings
Hollak et al. [19]	R	32 type 1 GD patients	Marked increased in chitotriosidase activity in 30 of 32 symptomatic type 1 GD patients. A decline in chitotriosidase activity was observed over a 50-week treatment period in 4 patients treated with ERT
Vellodi et al. [20]	R	28 pediatric GD patients treated with ERT	Compared with ACE and acid phosphatase, chitotriosidase activity showed the steepest negative slope over time, with the highest variation between patients and the smallest residual variance. ACE and chitotriosidase were strongly correlated within patient cohorts, whereas acid phosphatase was not correlated well with either ACE or chitotriosidase and had the largest residual variance
Guo et al. [24]	R	504 GD patients 385 healthy controls 205 patients with other lysosomal disorders	Marked elevation of chitotriosidase activity was specific for GD; none of the other lysosomal disorders showed elevation of plasma chitotriosidase activity as high as GD
Boot et al. [30]	P	55 type 1 GD patients 36 healthy controls	Plasma levels of CCL18 are markedly increased in symptomatic patients with GD and can act as an alternative surrogate disease marker. Monitoring of plasma CCL18 levels may be useful in determining therapeutic efficacy, especially in GD patients with deficient chitotriosidase activity
Chang et al. [29]	R	132 GD patients	Chemokine CCL18 represents an alternative option for patients with chitotriosidase deficiency for disease monitoring
Van Breemen et al. [32]	P	49 type 1 GD patients 39 healthy controls	An increase in plasma MIP-1β levels was associated with skeletal disease in Gaucher patients. Effective therapy decreased plasma levels of both MIP-1α and MIP-1β. High plasma MIP-1β (> 85 pg/ml) was observed in patients with ongoing skeletal disease despite 5 years of ERT
Danilov et al. [33]	P	Blood, spleen, and liver samples from GD patients and healthy controls	ACE activity and conformation in plasma and spleen samples from patients with GD differs from controls

ACE, angiotensin-converting enzyme; CCL18, chemokine [C-C motif] ligand; ERT, enzyme replacement therapy; GD, Gaucher disease; MIP-1 beta, macrophage inflammatory protein 1 beta; P, prospective; R, Retrospective

### Lyso-Gb1 as a specific and sensitive biomarker at diagnosis and clinical presentation

The need for a more reliable biomarker of GD activity and disease progression led to identifying the deacylated form of accumulated glucosylceramide, lyso-Gb1. Lyso-Gb1 is a direct metabolite of GBA and may play an essential role in disease-related pathology. Elevation of lyso-Gb1 was first reported in the grey matter of the brain and cerebellum of neuronopathic GD (type 2 and 3) patients, giving rise to the debate of its potential neurotoxic role [34]. Lyso-Gb1 was also detected in other organs, including the spleen and liver, in patients with type 1, 2, and 3 GD [35]. Mistry and colleagues first assessed a lyso-Gb1–based mechanism of skeletal disease in GD1 patients in a murine model with *GBA1* gene deletion, which showed the development of severe osteoporosis due to the accumulation of both lyso-Gb1 and glucosylceramide in osteoblasts, inhibiting protein kinase C and bone formation [36]. The role of lyso-Gb1 as a biomarker in GD patients in the studies discussed below can be found in Table 2.

**Table 2:** Lyso-Gb1 as specific and sensitive biomarker at diagnosis and clinical presentation

References	Study design	Population	Lyso-Gb1 measurment method	Key findings
Dekker et al. [37]	P, M	64 GD1 34 GD carriers 28 healthy controls	LC/MS/MS	In plasma of all GD1 patients, lyso-Gb1 was increased on average > 200-fold (15.6–1035 nM, median 230.7 nM), while only trace amounts of lyso-Gb1 were present in plasma of control subjects. Plasma lyso-Gb1 levels were not significantly increased in GD carriers. Plasma lyso-Gb1 levels, were significantly correlated with other plasma markers of Gaucher cells at diagnosis, including CCL18 and chitotriosidase, but not with MIP-1β. Lyso-Gb1 values were also associated with disease severity, mainly liver volume and bone mineral density
Rolfs et al. [38]	R, single center	98 GD patients 13 GD carriers 148 healthy controls 262 patients with other lysosomal storage disorders	HPLC–MS/MS	Elevated levels of lyso-Gb1 > 12 ng/ml were identified in GD patients but not in healthy controls, GD carriers, and patients with other lysosomal storage disorders. Lyso-Gb1 was more sensitive and specific than chitotriosidase and CCL18 at diagnosis based on a 12 ng/ml cut-off, which was established with an ideal sensitivity and specificity of 100% in 521 analyzed samples
Murugesan et al. [39]	P	169 GD1 41 healthy controls	LC/MS/MS	Lyso-Gb1 levels were increased by > 200-fold in untreated patients with GD1 compared with healthy controls (180.9 ng/mL versus 1.5 ng/mL). Patients with GD1 and healthy controls were distinguished by a cut off of 4 ng/mL, both with a sensitivity and specificity of 100%. Plasma lyso-Gb1 values between patients with GD1 and healthy controls did not overlap
Chipeaux et al. [40]	P, M	15 GD1 11 healthy controls	UHPLC-MS/MS	Lyso-Gb1 was one to two orders of magnitude higher in both plasma and RBCs of patients with GD1 compared with healthy controls
Tylki-Szymanska et al. [41]	R	64 GD patients	DBS	The variable "disease biomarker level" was dependent of the binary variable "treated with ERT or not" and independent of "disease type", "splenectomized or not", and "heterozygous for 24-bp duplication for *CHIT1* variant" or "*CHIT1* wild type"
Irùn et al. [42]	R	47 GD patients 19 GD carriers 42 healthy controls 37 patients with other lysosomal lipidoses	LC/MS/MS	Only GD patients displayed lyso-Gb1 levels above 5.4 ng/mL at diagnosis. Plasma lyso-Gb1 was significantly correlated with the biomarkers, chitotriosidase activity and CCL18 (both p < 0.001), but not with clinical parameters related to disease burden
Hurvitz et al. [48]	R	35 mild GD1 34 severe GD1 12 type 3 GD	DBS	Significantly higher lyso-Gb1 levels were identified at baseline in children with more symptomatic disease (i.e., thrombocytopenia, anemia, and hepatosplenomegaly) who subsequently underwent ERT compared with untreated children (p = 0.0003) and, at the last visit, in children with severe GD1 than those with mild GD1 (p = 0.009) In the total patient population, lyso-Gb1 correlated significantly with platelet count (p < 0.0001) and hemoglobin levels (p = 0.003), but not with liver and spleen volume, child’s age, and weight
Saville et al. [49]	R	12 non-neuronopathic GD 11 neuronopathic GD 156 controls 3 GD carriers 37 other-IMD	DBS	Higher median lyso-Gb1 concentrations were detected in DBS from non-neuronopathic GD and neuronopathic GD patients compared with controls (1.65 and 7.07 vs. < 0.06 pmol/spot, respectively). Significantly higher plasma lyso-Gb1 levels were identified in patients with a neuronopathic phentoype than in those with a non-neuronopathic phenotype (p < 0.0001). Elevated plasma lyso-Gb1 levels (70 nmol/L) were detected in a 1-day-old neonate, with an affected older sibling, who was subsequently confirmed as homozygous for N370S. Plasma lyso-Gb1 concentrations of 1,070–2,620 nmol/L were detected in 4 neuronopathic GD patients aged < 20 days old
Stiles et al. [50]	Case report	Case 1: 7-year-old male with GD diagnosed prenatally Case 2: 9-year-old male with GD diagnosed at 5 years of age due to a positive family history	UPLC-MS/MS	Lyso-Gb1, as a key biomarker, is useful in guiding treatment initiation Case 1 had no outward signs of disease such as pain, fracture, or bleeding, however, regular follow-up appointments from 3 years of age identified persistent hepatosplenomegaly and marked elevations in chitotriosidase and lyso-Gb1 levels. ERT was recommended at 7.4 years of age, with marked reduction observed in biomarker values after 3 months of treatment For Case 2, evidence of disease burden (pain, low bone density, and borderline low platelets) alongside elevated chitotriosidase and lyso-Gb1 levels supported the decision to initiation treatment with ERT at 9.2 years of age, with marked reductions in biomarker levels observed after 5 months of treatment
Cozma et al. [51]	R	19 GD patients treated with ERT	DBS	Lyso-Gb1 was reliably detected in DBS samples over a 3-year period. After an involuntary treatment break, the separation of lyso-Gb1 levels “under treatment” versus “not under treatment” was identified with high sensitivity and specificity

*CHIT1*, Chitotriosidase gene; DBS, dry blood spot; ERT, enzyme replacement therapy; GD, Gaucher disease; GD1, type 1 GD; HPLC–MS/MS, high performance liquid chromatography tandem mass spectrometry; IMD, inherited metabolic disorder; LC/MS/MS, liquid chromatography tandem mass spectrometry; Lyso-Gb1, glucosylsphingosine; M, multicenter; P, prospective; R, Retrospective; RBC, red blood cells; UHPLC-MS/MS, ultra-high pressure liquid chromatography tandem mass spectrometry; UPLC-MS/MS, ultraperformance liquid chromatography-tandem mass spectrometry; vs., versus

Elevated plasma lyso-Gb1 levels were demonstrated in non-neuronopathic GD1 patients compared with obligate carriers of the GD mutation and healthy subjects, and were associated with disease severity, mainly liver volume and bone mineral density [37].

The specificity of lyso-Gb1 as a biomarker for GD was established in 2013 [38], with pathological levels identified in GD patients but not in healthy controls, GD carriers, and patients with other lysosomal storage disorders. Furthermore, lyso-Gb1 was more sensitive and specific than chitotriosidase and CCL18 at diagnosis based on a 12 ng/ml cut-off. A separate study also confirmed lyso-Gb1 as a key biomarker of GD at diagnosis, although a cut-off of 4 ng/ml distinguished GD patients versus healthy controls [39]. Lyso-Gb1 levels also correlated with established biomarkers and clinical indicators of disease burden, including chitotriosidase, CCL18, liver and spleen volume, and splenectomy (all p ≤ 0.01). The superiority of lyso-Gb1 as a biomarker of GD in plasma and red blood cells (RBCs), compared with glucosylceramide, sphingosine, and sphingosine-1-phosphate, was determined using ultra-high pressure liquid chromatography-tandem mass spectrometry (UHPLC-MS/MS) in a prospective multicenter study [40].

Interestingly, both lyso-Gb1, measured with dry blood spot (DBS) mass spectrometry, and chitotriosidase levels were found to be independent of disease type (neuronopathic versus non-neuronopathic) and splenectomy status [41].

Compared with chitotriosidase and CCL18, only lyso-Gb1 levels above 5.4 ng/mL were identified at diagnosis of GD patients with 100% sensitivity and specificity [42]. Furthermore, plasma lyso-Gb1 correlated significantly with chitotriosidase activity and CCL18, but not with clinical parameters related to disease burden (i.e., platelet count, hemoglobin, spleen and liver volume, or disease severity).

A pathophysiological role of lyso-Gb1 in GD was suggested in a long-term infusion model in genetically normal mice [43]. In this study, continuous systemic subcutaneous administration of lyso-Gb1 elevated lyso-Gb1 levels > 500-fold compared with vehicle-treated mice, reflecting concentrations seen in severely affected untreated GD patients. Lyso-Gb1 accumulated in peripheral tissues, and the mice developed hematological and visceral symptoms, namely reduced hemoglobin and hematocrit levels and increased spleen size, together with a slight inflammatory tissue response after 8-weeks of treatment. Elevated lyso-Gb1 levels at baseline were also identified in treatment-naïve GD1 patients in the open-label phase 2 study (NCT00358150) [44], and the phase 3 ENGAGE trial (NCT00891202) [45], with correlations observed between high baseline lyso-Gb1 levels and disease severity, mainly spleen and liver volume and hemoglobin levels, prior to eliglustat therapy [46].

Discordant results have been reported regarding the *GBA1* mutation status of GD patients, mainly *N370S* and *L444P* in 70% of cases, and its association with plasma lyso-Gb1 levels [37, 38, 47].

Lyso-Gb1 as a biomarker was also evaluated in the pediatric population, with outcomes showing significant correlations between lyso-Gb1 levels and disease severity [48]. Specifically, significantly higher lyso-Gb1 levels were identified at baseline in children with more symptomatic disease (i.e., thrombocytopenia, anemia, and hepatosplenomegaly) who subsequently underwent ERT compared with untreated children (p = 0.0003) and, at the last visit of treated patients, in children with severe GD1 compared to those with mild GD1 (p = 0.009).

Lyso-Gb1 in DBS may hold promise as a screening tool in newborns and be beneficial to monitor disease course, with significantly higher plasma lyso-Gb1 levels detected in non-neuronopathic and neuronopathic GD patients compared with controls and in neuronopathic GD patients compared with non-neuronopathic GD patients [49]. Lyso-Gb1 was also beneficial as an early indicator of disease progression in two treatment-naïve pediatric patients with GD1 supporting the decision to initiate treatment despite no outward signs of disease in one patient and only mild symptoms in the second [50]. Lyso-Gb1 also shows clinical utility in monitoring treatment response in GD patients [51]. This study validated lyso-Gb1 quantification in DBS samples as a valid measurement and demonstrated a general trend of decreasing lyso-Gb1 levels with continuous ERT over 25-months. Moreover, rising lyso-Gb1 levels identified during a forced treatment break reliably flagged the loss of therapeutic effect. These results suggest that lyso-Gb1 as a biomarker could be used to identify issues with treatment at an early stage and before clinical consequences arise.

### Lyso-Gb1 and Parkinson’s disease

Parkinson’s disease (PD) is a progressive neurodegenerative disease, with aggregated α-synuclein and Lewy body formation that represents an integral component of disease pathogenesis [52–54]. Given the increased risk for PD in both GD patients and carriers [55], with between 7 and 20% of patients with PD carrying a *GBA* mutation [56], accumulation of glucosylceramide and its metabolites represent potential targets for neurodegenerative treatment.

A recent study in a murine model with *GBA1* deficiency demonstrated the role of downstream glucosylceramide metabolites, namely lyso-Gb1, sphingosine, and sphingosine-1-phosphate, in promoting α-synuclein aggregation and toxicity [57]. Furthermore, the accumulation of lyso-Gb1 in the mouse brain was confirmed, with acid ceramidase and GBA2 proposed as potential new therapeutic targets for the prevention and acute treatment of *GBA*-associated PD. In addition, a prodromal mouse model of PD confirmed the impairment of dopaminergic neuron function in mice with a null *GBA* allele associated with lyso-Gb1 accumulation [58]. Finally, post-mortem brain autopsies of patients with PD or dementia with Lewy bodies demonstrated a direct correlation between α-synuclein levels and lyso-Gb1 in humans [59].

### Lyso-Gb1 role in multiple myeloma

GD is commonly associated with persistent age-related monoclonal and polyclonal gammopathy and an increased incidence of clonal B-cell proliferation such as non-Hodgkin lymphoma and multiple myeloma. However, the mechanism of tumorigenesis in GD remains uncertain. One hypothesis suggests that chronic inflammation with alternatively activated macrophages that secrete pro-inflammatory cytokines and chemokines, mainly interleukin-6 and interleukin-10 due to prolonged accumulation of glycosphingolipids, stimulate the clonal expansion of B lymphocytes and plasma cells [60–64].

Increased concentrations of lyso-Gb1 were identified in murine models with *GBA1* gene deficiency, which was associated with monoclonal gammopathy in most cases [65]. The sporadic development of both B cell lymphomas and multiple myeloma could suggest a bioactive role of glycosphingolipids that could hypothetically stimulate the proliferation of mature B lymphocytes and plasma cells. These results are consistent with a separate study, which demonstrated reduced malignant lymphoproliferation together with decreased beta-glucosylceramide and deacylated glycosphingolipid levels in eliglustat-treated Gaucher mice [66].

In monoclonal B-cell pathogenesis, lyso-Gb1 influenced antigen-specific type II natural killer T cells that stimulate T-follicular helper phenotype leading to immune dysfunction [67]. Further studies confirmed that monoclonal immunoglobulins from patients affected by monoclonal gammopathy in GD were specific against lyso-Gb1 and that lyso-Gb1 mediates the activation of B lymphocytes and plasma cells [67, 68].

### Lyso-Gb1 levels in Red Blood Cells

Higher levels of several sphingolipids, including lyso-Gb1, have been found in RBCs from untreated GD patients than in healthy controls [40], while ERT treatment significantly decreased lyso-Gb1 levels in RBCs [69]. Sphingolipid accumulation in RBCs may explain symptoms like anemia and ischemic events; however, its role in the pathophysiology of GD is uncertain.

### Enzyme replacement therapy: role in the variation of plasma lyso-Gb1 levels

A reliable biomarker is crucial not only for monitoring disease progression, but also to assess treatment response. Therefore, it is imperative to understand the variation of plasma lyso-Gb1 levels in untreated and treated patients with GD and differences between plasma lyso-Gb1 and plasma chitotriosidase levels during standard ERT. Detailed descriptions of the ERT studies and lyso-Gb1 levels described below can be found in Table 3.

**Table 3:** Enzyme replacement therapy: role in the variation of lyso-Gb1 plasma levels

References	Study design	Population	Key findings
Dekker et al. [37]	P, M	64 GD1 patients	Marked reduction of lyso-Gb1 levels were observed in most GD1 patients receiving ERT (imiglucerase or alglucerase)
Rolfs et al. [38]	R, single center	19 GD patients	Significant and rapid reductions in lyso-Gb1 levels over time after commencing ERT (lyso-Gb1 levels ranged from 50 to 250 ng/ml prior to ERT), with the most marked reductions occurring immediately after the start and within the first 6 months of ERT, with lyso-Gb1 values below 50 ng/ml achieved in most patients
Elstein et al. [47]	R	22 treatment-naïve GD patients 21 GD patients previously treated with imiglucerase	Lyso-Gb1 levels decreased 82.7% in treatment-naïve patients (from 323.2 ± 29.9 ng/mL at baseline to 60.4 ± 11.3 ng/mL at week 209) and 52.0% in previously-treated patients (from 81.8 ± 15.8 ng/mL at baseline to 52.8 ± 15.2 ng/mL at week 161) In treatment-naïve patients, decreasing lyso-Gb1 levels were significantly correlated with increasing platelet counts at weeks 13, 25, and 53 (p = 0.0112, p = 0.0010, and p = 0.0171, respectively) and with decreasing spleen volumes at weeks 25, 101, and 209 (p = 0.0235, p = 0.0318, and p = 0.0093, respectively). No statistically significant correlations were observed between lyso-Gb1 levels and platelets counts or spleen volumes in previously-treated patients
Tylki-Szymańskaa et al. [41]	R	64 GD patients	The variable "disease biomarker level" was found dependent of the binary variable "treated with ERT or not"
Hurvitz et al. [48]	R	40 pediatric GD patients	Lyso-Gb1 levels were inversely correlated with platelet counts in untreated children (p = 0.002) and with hemoglobin levels in treated children (p = 0.01). Lyso-Gb1 levels increased in almost 50% of untreated children during follow-up, more commonly in younger children. The increase in lyso-Gb1 levels while receiving ERT, seen in 8 children, was partly associated with non-compliance and weight gain (> 15%) without dose adjustment
Murugesan et al. [39]	P, M	169 GD1 patients under treatment (155 on ERT and 14 on eliglustat)	The long-term response of chitotriosidase and lyso-Gb1 to ERT, calculated as mean elevations of ULN, showed chitotriosidase was increased 29.2xULN at year 1 and decreased to 15.6xULN after 3-years treatment, whereas lyso-Gb1 was increased 62.8xULN at year 1 and decreased to 20.2xULN by year 3
Arkadir et al. [70]	R	25 GD1 patients with homozygosis *N370S* treated with ERT imiglucerase (n = 4) velaglucerase alfa (n = 17) taliglucerase alfa (n = 4)	Plasma lyso-Gb1 levels decreased markedly in the whole cohort independent of drug type, along with an increase in both hemoglobin and platelet counts and a decrease in spleen volume The decay in lyso-Gb1 levels after ERT followed an exponential curve. Determination of the half-life for normalization of plasma lyso-Gb1 levels and spleen volume showed that these were half-normalized after 15.4 months and 112 months, respectively. Notably, the calculated half-life of lyso-Gb1 was markedly shorter in patients treated with velaglucerase alfa than in those receiving imiglucerase or taliglucerase alfa (14.7 months vs. 17.6 months), a pattern also observed for the calculated decay half-life of spleen reduction (118 months vs. 138 months)
Dinur et al. [71]	R	135 adult patients with GD1 treated with ERT imiglucerase (n = 41) velaglucerase alfa (n = 73) taliglucerase alfa (n = 21)	Longitudinal observations showed decreasing lyso-Gb1 values over time compared with starting values, with values plateauing at around 100 months (approximately 8 years) on treatment. A large inter- and intra-individual variation in lyso-Gb1 levels was identified for all three ERTs

ERT, enzyme replacement therapy; GD, Gaucher disease; GD1, type 1 GD; Lyso-Gb1, glucosylsphingosine; M, multicenter; P, prospective; R, Retrospective; ULN, upper limit of normal

ERT rapidly reduced plasma lyso-Gb1 levels in most GD1 patients compared with baseline, with comparable reductions in plasma chitotriosidase and CCL18 levels, although 5 GD1 patients had a poor response in plasma lyso-Gb1 levels that did not coincide with the reductions in chitotriosidase and CCL18 [37]. Plasma lyso-Gb1 levels also decreased in three type 3 GD patients homozygous for the L444P mutation treated with ERT in combination with SRT, with a comparable effect on plasma chitotriosidase levels. Decreased lyso-Gb1 levels were also observed during ERT in a separate study, with the most pronounced reduction occurring within the first 6 months of ERT [38].

The change in plasma lyso-Gb1 levels might reflect clinical response to ERT treatment [47]. In a retrospective analysis from phase 3 clinical studies of GD1 patients treated with velaglucerase alfa, baseline plasma lyso-Gb1 levels decreased over time in both treatment-naïve patients and those previously treated with imiglucerase, with a more pronounced response in treatment-naïve patients. In treatment-naïve patients, plasma lyso-Gb1 levels were significantly correlated with increased platelet counts, albeit not past week 53, and reduced spleen volume. These correlations were not demonstrated in previously-treated patients.

ERT treatment substantially modified the distribution of both chitotriosidase and lyso-Gb1 levels in patients with GD, with levels following a normal distribution only in untreated patients [41]. In addition, a linear correlation between plasma chitotriosidase activity and lyso-Gb1 levels was identified at treatment start and with increasing ERT dose, except for patients with elevated disease burden treated with high dose ERT (> 35 U/kg).

Hurvitz and colleagues evaluated the impact of ERT treatment in GD pediatric patients with symptomatic disease, including hematological and visceral abnormalities [48]. There was a more significant decrease in lyso-Gb1 levels from baseline to the last measurement in treated patients with pretreatment measurements than in those with both measurements taken while on therapy. Interestingly, lyso-Gb1 levels increased in 8 children treated with ERT; however, this was likely due to weight gain (> 15%) without dose adjustment and lack of compliance [48].

Plasma lyso-Gb1, as a key biomarker of GD, was demonstrated by the long-term response of chitotriosidase and lyso-Gb1 to ERT, calculated as mean elevations of the upper limit of normal (ULN) [39]. Specifically, lyso-Gb1 levels showed a more striking and rapid response than chitotriosidase, with the average fold elevation in lyso-Gb1 levels by ULN twice compared to chitotriosidase after 1 year of treatment, and lyso-Gb1 levels decreased to one third by year 3, whereas chitotriosidase levels were halved. Given that lyso-Gb1 is directly involved in the pathological pathway of GD, and may therefore more accurately represent residual whole-body GD activity, lyso-Gb1 may be the biomarker of choice to evaluate disease burden and monitor treatment response, compared with chitotriosidase, which is specifically secreted by activated macrophages.

Lyso-Gb1 was also shown to be a reliable rate biomarker in quantifying clinical response to ERT in GD patients [70]. Moreover, longitudinal observations of lyso-Gb1 levels in individual patients treated with ERTs showed decreasing values over time compared with starting values, with values plateauing at around 100 months (approximately 8 years) of treatment [71].

### Substrate reduction therapy: role in the variation of plasma lyso-Gb1 levels

Although ERT has been considered the gold standard for GD treatment over the last 30 years, the SRTs, miglustat and eliglustat, are also important therapeutic options.

Miglustat was approved in 2002 in the European Union and is indicated to treat adult patients with mild-to-moderate GD1 who are unsuitable to receive ERT [13]. Eliglustat, which has been available since 2015 and is approved in the European Union for the long-term treatment of adult GD1 patients with extensive, intermediate, or poor CYP2D6-metabolizer phenotypes (> 90% of patients), decreases the rate of glucosylceramide production by inhibiting the enzyme, glucosylceramide synthase [14, 72, 73]. Table 4 summarizes the studies described below regarding the variation in plasma lyso-Gb1 levels in GD patients treated with SRT, including those who switched from long-term ERT.

**Table 4:** Substrate reduction therapy: role in the variation of plasma lyso-Gb1 levels

References	Study design	Population	Key findings
Lukina et al. [44]	P, M	26 untreated GD1 patients, all with splenomegaly, thrombocytopenia, and/or anemia prior to treatment	Eliglustat 50 or 100 mg twice daily decreased plasma lyso-Gb1 levels from baseline to year 8 (624 ng/mL vs. 47.6 ng/mL), a percent reduction of 92%, with marked reductions at 1 year, which continued through 8 years of eliglustat therapy
Mistry et al. [45]	P, M	40 untreated GD patients	Despite elevated serum lyso-Gb1 levels in all patients at baseline (median value 61-fold above the ULN), median decreases in serum lyso-Gb1 levels of 59% and 84% were observed after 9 months (n = 37) and 4.5 years (n = 10) of eliglustat treatment, respectively
Peterschmit et al. [46]	P, M	62 untreated GD1 patients	The decrease in lyso-Gb1 levels correlated with improvements in baseline clinical parameters after eliglustat treatment (spleen, liver, hemoglobin, and platelets; all p < 0.05)
Murugesan et al. [39]	P, M	169 GD1 patients under treatment (155 on ERT and 14 on eliglustat)	Significantly lower levels of lyso-Gb1 were identified in patients treated with eliglustat than those receiving ERT (p < 0.001). Clinical indicators independently predictive of plasma lyso-Gb1 levels by multi-variate regression analysis were age (p < 0.001), serum chitotriosidase (p < 0.001), serum CCL18 (p = 0.001), splenectomy (p = 0.02), and treatment with eliglustat (p < 0.001). Interestingly, lyso-Gb1 levels were decreased to a greater extent among patients receiving eliglustat (9 patients) than those receiving ERT (47 patients) in comparable patient groups identified by propensity score matching
Smid et al., 2016 [74]	R	17 treatment-naïve or ERT experienced GD1 patients treated with eliglustat (n = 6) miglustat (n = 9) ERT (n = 4)	Biochemical markers (chitotriosidase and lyso-Gb1) respond comparably in treatment-naïve patients receiving eliglustat treatment (n = 4) or ERT (n = 4), whereas the response was lower in miglustat-treated patients (n = 2). Specifically, in treatment-naïve patients, a median decrease in chitotriosidase levels of 89% [range 77–98], 88% [78–92], and 37% [29–46] were identified for eliglustat, ERT, and miglustat-treated patients respectively; for lyso-Gb1 levels this was 86% [78–93], 78% [65–91], and 48% [46–50], respectively
Kleytman et al. [75]	Real-world, longitudinal study	38 GD1 patients on long-term ERT for ≥ 2 years (mean 14.3 years; range 2 to 26 years) who had reached therapeutic goals before switching to eliglustat SRT (mean 3.1 years; range 5 months to 7 years)	Plasma lyso-Gb1 levels decreased significantly from 63.7 ng/ml (95% CI, 37.6–89.8) to 26.1 ng/ ml (95% CI, 15.7–36.6) (p < 0.0001) on eliglustat. In addition, 15 patients showed reductions of serum lyso-Gb1 to near normal levels (normal distribution of plasma lyso-Gb1 is 3.3 ng/ml) Serum chitotriosidase levels also fell from 1,137 nmol/ml/h (95% CI, 145–2129) to 467 nmol/ml/h (95% CI, 209.9–724) (p = 0.002)

ERT, enzyme replacement therapy; GD, Gaucher disease; GD1, type 1 GD; Lyso-Gb1, glucosylsphingosine; M, multicenter; P, prospective; R, Retrospective; ULN, upper limit of normal

Marked reductions in plasma lyso-Gb1 levels were observed during the first year of eliglustat therapy in treatment-naïve GD1 patients, with reduced levels maintained over 4.5-year (the ENGAGE trial) [45] and 8-year [44] treatment periods and similar trends in biomarker response observed for chitotriosidase, CCL18, and glucosylceramide. Notably, decreased lyso-Gb1 levels correlated with improved clinical parameters of the spleen, liver, hemoglobin, and platelets (all p < 0.05), highlighting the clinical utility of lyso-Gb1 in disease monitoring [46].

The utility of lyso-Gb1 as a valid biomarker of treatment response in GD1 was demonstrated in GD1 patients over a 5-year treatment period, with lyso-Gb1 levels correlated with established biomarkers and clinical indicators of disease burden [39]. Interestingly, lyso-Gb1 levels decreased to a greater extent among patients receiving eliglustat (9 patients) than those receiving ERT (47 patients) in comparable patient groups identified by propensity score matching [39]. In a separate study, clinical response in chitotriosidase and lyso-Gb1 levels were comparable in treatment-naïve GD1 patients who received 2-years of eliglustat or ERT, whereas biomarker response was lower in miglustat-treated patients [74].

Finally, significant decreases in serum lyso-Gb1 levels were identified in therapeutically stable GD1 patients who switched from long-term ERT to eliglustat SRT, with near-normal levels restored in 15 patients [75]. In addition, significant decreases in serum chitotriosidase levels were observed.

### New insights

More recently, a simple and accurate method to determine lyso-Gb1 measurements in DBS samples was established as a useful tool for the screening and diagnosis of GD [76], while a separate study showed that lyso-Gb1 measured in DBS samples alongside whole-gene sequencing reliably diagnosed GD, although lyso-Gb1 levels did not differentiate between heterozygous GBA1 carriers and wild type [77]. Nonetheless, Dinur and colleagues proposed a paradigm change for screening patients suspected to have GD based on an analysis of lyso-Gb1 measurements and *GBA1* mutation analyses in DBS [77].

## Conclusions

GD is a rare genetic disorder that is difficult to diagnose and manage. Biomarkers are valuable tools to monitor disease progression and treatment response. Lyso-Gb1 is the most promising biomarker of GD, as demonstrated by its reliability in reflecting disease burden and monitoring treatment response. Furthermore, lyso-Gb1 has an important role in the pathogenetic mechanism of PD due to its cerebral accumulation, and in B-cell lymphoproliferative disorders, such as multiple myeloma, due to humoral immunity dysregulation by chronic antigenic stimulation. Early treatment intervention in GD patients could reduce its accumulation, thus hypothetically lowering the risk of developing neurodegenerative disease or multiple myeloma.

## Data Availability

All data analyzed during this study are included in this published article.

## Abbreviations

ACE: Angiotensin-converting enzyme
CCL18: Chemokine [C-C motif] ligand
DBS: Dry blood spot
ERT: Enzyme Replacement Therapy
GBA: Acid β-glucosidase
GD: Gaucher disease
LC/MS/MS: Liquid chromatography tandem mass spectrometry
Lyso-Gb1: Glucosylsphingosine
MIP-1β: Macrophage inflammatory protein 1 beta
PD: Parkinson’s disease
SRT: Substrate Reduction Therapy
TRAP5b: Tartrate-resistant acid phosphatase 5b
UHPLC-MS/MS: Ultra-high pressure liquid chromatography-tandem mass spectrometry
ULN: Upper limit of normal
